# A Hybrid Approach to Detect Driver Drowsiness Utilizing Physiological Signals to Improve System Performance and Wearability

**DOI:** 10.3390/s17091991

**Published:** 2017-08-31

**Authors:** Muhammad Awais, Nasreen Badruddin, Micheal Drieberg

**Affiliations:** 1 Centre for Intelligent Signal and Imaging Research, Universiti Teknologi PETRONAS (UTP), Seri Iskandar 32610, Malaysia; 2 Department of Electrical, Electronic, and Information Engineering Guglielmo Marconi, University of Bologna, Bologna 40126, Italy; muhammad.awais2@unibo.it; 3 Department of Electrical and Electronics Engineering, Universiti Teknologi PETRONAS (UTP), Seri Iskandar 32610, Malaysia; mdrieberg@utp.edu.my

**Keywords:** EEG, ECG, HRV, SVM, driver drowsiness detection, channel reduction

## Abstract

Driver drowsiness is a major cause of fatal accidents, injury, and property damage, and has become an area of substantial research attention in recent years. The present study proposes a method to detect drowsiness in drivers which integrates features of electrocardiography (ECG) and electroencephalography (EEG) to improve detection performance. The study measures differences between the alert and drowsy states from physiological data collected from 22 healthy subjects in a driving simulator-based study. A monotonous driving environment is used to induce drowsiness in the participants. Various time and frequency domain feature were extracted from EEG including time domain statistical descriptors, complexity measures and power spectral measures. Features extracted from the ECG signal included heart rate (HR) and heart rate variability (HRV), including low frequency (LF), high frequency (HF) and LF/HF ratio. Furthermore, subjective sleepiness scale is also assessed to study its relationship with drowsiness. We used paired *t*-tests to select only statistically significant features (*p* < 0.05), that can differentiate between the alert and drowsy states effectively. Significant features of both modalities (EEG and ECG) are then combined to investigate the improvement in performance using support vector machine (SVM) classifier. The other main contribution of this paper is the study on channel reduction and its impact to the performance of detection. The proposed method demonstrated that combining EEG and ECG has improved the system’s performance in discriminating between alert and drowsy states, instead of using them alone. Our channel reduction analysis revealed that an acceptable level of accuracy (80%) could be achieved by combining just two electrodes (one EEG and one ECG), indicating the feasibility of a system with improved wearability compared with existing systems involving many electrodes. Overall, our results demonstrate that the proposed method can be a viable solution for a practical driver drowsiness system that is both accurate and comfortable to wear.

## 1. Introduction

Safety has received an unprecedented amount of research attention in recent years, particularly in relation to transportation. Transportation safety research has typically focused on increasing the level of safety for drivers and their surroundings, aiming to reduce the high number of road accidents. Driving is a complex task that requires mental and physical attention and alertness to perform effectively. Recent studies [1,2] have shown that drowsiness at the wheel is a major contributing factor in road accidents, causing a large number of deaths, serious injuries and financial losses. A study conducted by the National Highway Traffic Safety Administration (NHTSA) in the United States reported 416,000 drowsiness-related accidents from 2005 to 2009 [1]. Another study conducted by the Centre for Accident Research and Road Safety in Queensland, Australia [2], reported that drowsiness was one of the leading factors contributing to road accidents, among other factors including speeding and drinking. In addition, it was reported that 20–30% of all road deaths are the direct result of driver drowsiness [2]. Moreover, the study reported that the likelihood of crashes is greater on long straight roads, highlighting the risks of drowsiness. Furthermore, due to the subjective nature of accident reporting, many crashes are not verified by the police, suggesting that accidents due to drowsiness may be substantially more common than has been estimated in the past [2]. 

The term drowsiness typically refers to a state of sleepiness and apathy that leads to the tendency to fall asleep [3]. The human sleep cycle is divided into three categories: wakefulness, non-rapid eye movement (NREM) and rapid eye movement (REM) [4]. The first category is a state of consciousness in which a person is completely alert and can perform physical and mental tasks while maintaining attention. The second category, NREM, is divided into three stages; stage one is related to drowsiness, while stages two and three are associated with light sleep and deep sleep states, respectively. Drowsiness is an intermediate state between sleepiness and wakefulness. It is a state of reduced attention and vigilance towards any tasks being performed [5]. Thus, drowsiness is dangerous in driving situations, where a driver’s loss of attention and increased response time can cause road accidents resulting in serious injuries and deaths. The major contributing factors to drowsiness are long working hours, lack of sleep, and continuous driving [6].

## 2. Related Work

Recent approaches for detecting driver drowsiness can be divided into four main categories: vehicle-based measures, subjective measures, driver behavioral measures and physiological measures [3]. 

### 2.1. Vehicle-Based Measures

Vehicle-based measures provide an assessment of driving performance by analyzing the capabilities of the driver and the way they control a vehicle. These measures include steering wheel movement, speed variability and standard deviation of lateral position [7,8,9,10]. A major limitation of these measures is that variations in steering wheel movement or standard deviation of lateral position typically occur only during the later stages of drowsiness when it is too late to prevent an accident from occurring. Furthermore, these measures are highly dependent upon road geometry and are effective in a limited range of conditions. When exposed to real environments with substantial variation, these systems often fail to function effectively [11,12]. 

### 2.2. Subjective Measures 

The second category is subjective measures, in which drivers themselves estimate their level of sleepiness. The commonly used sleepiness scales are: the Stanford Sleepiness Scale (SSS) [13,14] and the Karolinska Sleepiness Scale (KSS) [15]. A major drawback of these types of measure is the infeasibility of implementation in real-world driving conditions due to their subjective nature. Asking a driver to rate their arousal level may stimulate alertness, thus biasing the ratings. In addition, variations in self-rated drowsiness can also be caused by stress, or the influence of alcohol [3,12,16], which may not be the case for drowsiness. Some studies have also shown that this kind of measures demonstrates low correlation and disassociation with objective measures [17,18].

### 2.3. Driver Behavioral Measures

The third type of measure, driver behavioral measures, is primarily focused on the driver’s ability to concentrate on driving, typically obtained through behavioral parameters (e.g., driver’s head movements, eye blink duration, eye blink frequency, yawning, and facial expression) [19,20,21,22,23,24] mostly detected by image processing techniques. Although driver behavioral measures are non-intrusive, such measures have not been found to be reliable for the detection of drowsiness, because their detection capabilities are strongly affected by variations in environmental factors and driving conditions [25,26,27]. For instance, change in lighting conditions inside or outside the vehicle during the day/night and use of glasses (affecting the measurement of eye blink duration and frequency) by the subject will decrease the detection capabilities of image processing based systems [3]. Furthermore, studies of these measures have typically tested non-drowsy subjects by instructing them to mimic drowsiness-related behaviors, such as yawning and closing the eyes [3].

### 2.4. Driver Physiological Measures

The last category of measures involves the use of physiological measurements for the detection of driver drowsiness. Physiological signals are considered to provide an accurate measure of drowsiness because of their strong relationship with driver fatigue [3,4,5,6]. Electroencephalography (EEG) is considered one of the most reliable methods for drowsiness detection. Raw data acquired from EEG electrodes can be divided into different frequency bands after preprocessing, which involves artifact removal and filtering. Commonly used frequency bands include the delta (0.5–4 Hz), theta (4–8 Hz), alpha (8–13 Hz), beta (13–30 Hz), and gamma (greater than 30 Hz) bands [3]. Several previous studies have used various time and frequency features of the EEG signal to detect drowsiness [27,28,29,30,31]. Other studies have also used ECG for the detection of drowsiness, and HRV derived from ECG has been used to differentiate between alertness and drowsiness [32,33,34,35]. Although these approaches have proven relatively effective for examining physiological and cognitive states in humans, issues with wearability, particularly for EEG devices, have limited the feasibility of using these systems in real-world driving conditions. Lin et al. [26] made an effort to develop a real-time EEG based drowsiness detection system in by reducing the number of EEG electrodes to three. However, our idea was to further reduce the number of EEG sensors to make it more practical in real driving conditions, and to combine it with a single ECG sensor in order to improve the system’s performance.

Therefore, the main contributions of this paper are twofold. Firstly, this study looks into the possibility of using features extracted from EEG and ECG to differentiate between alert and drowsy states. Secondly, it explores the potential for channel reduction while maintaining an acceptable level of accuracy. The present study proposes a hybrid approach for detecting driver drowsiness in a monotonous driving environment. The main objectives of this study are:

- to detect drowsiness using EEG and ECG alone, and combining both modalities to improve the performance of the system;
- to propose a channel reduction paradigm to reduce the number of electrodes and to increase the practicality of the proposed technique in real driving environments.

To the best of our knowledge, none of the existing systems are capable of detecting drowsiness by combining the features analyzed in this study (acquired from ECG and EEG). Furthermore, the channel reduction approach analyzed in this study is first of its kind in providing the optimal number of electrodes with an acceptable level of performance. Therefore, the present study sought to examine the performance of these physiological measures in detecting driver drowsiness. 

The rest of the article is organized as follows: Section 2 presents the methodology of the proposed system including the experimental set-up, studied population, and the subjective and physiological measures used in the study. Section 3 presents the results of the study, an assessment of the performance of integrating physiological measures and the feasibility of a channel reduction approach. Finally, Section 4 summarizes the research findings.

## 3. Materials and Methods for Inducing and Detecting Driver Drowsiness

### 3.1. Simulator-Based Driving Environment

**Figure 1:**
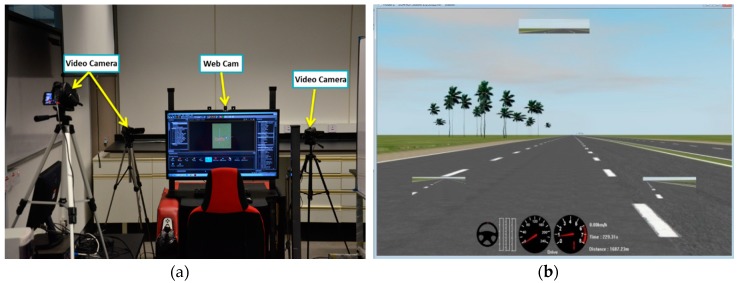
(**a**) Experimental setup to induce drowsiness; (**b**) view of a monotonous driving session.

Data collection was performed in a simulator-based driving environment, developed in collaboration with the Malaysian Institute of Road Safety Research (MIROS). An illustration of the experimental setup is shown in Figure 1a. Simulator-based environments were chosen over real driving conditions, because of the risks and ethical implications of allowing subjects to drive in a state of drowsiness. A monotonous driving environment was created as shown in Figure 1b to induce drowsiness in the driver naturally, as these concepts has been used in the literature [10,36].

### 3.2. Sample Population

A sample of 22 healthy subjects provided informed consent to participate in the study. All subjects were university students, aged between 18 and 35 years, free from any medication, serious head injuries or hearing impairments and had normal or corrected-to-normal vision. All subjects had at least two years of driving experience. Each subject gave written consent before the study began, and subjects were free to leave the experiment in any stage if they felt uncomfortable. This study received ethics approval from the Centre for Intelligent Signal and Imaging Research (CISIR) Research Ethics Committee of Universiti Teknologi PETRONAS (UTP).

### 3.3. Materials 

Physiological data collection was performed using an Enobio-20 channel device from Neuroelectrics [37]. The Enobio device follows the 10–20 international system and uses dry electrodes that transmit physiological data (both EEG and ECG data) to a computer via a Bluetooth connection. Wet electrodes are not recommended for long durations of recording, because they become dry after a relatively short time, typically requiring gel application to maintain good conductivity over the measurement period. This may interrupt the driving task and alert the driver. An ECG sensor was applied to the bottom of the neck to record the heart activity. The experimental setup included three video cameras and one webcam mounted on the dashboard to provide visual information to be used as ground truth for drowsiness-related events (Figure 1a). 

### 3.4. Data Collection

The data collection procedure was divided into three sessions, in addition to the briefing and debriefing sessions: a familiarization driving (FD) phase without a fixed duration, for the subject to become accustomed to the simulated driving environment; a 10-min Training Driving (TD) phase, in which the physiological data acquisition device was worn by the subject to check the comfort level; and, a Monotonous Driving (MD) phase. In the MD phase, subjects drove the car for 80 min in a highway scenario with a few other cars on the road, maintaining a maximum vehicle speed of 80 km/h, to induce drowsiness naturally [10]. 

### 3.5. Subjective Measure

The Karolinska Sleepiness Scale (KSS) [15] served as a subjective measure in this study. The KSS is the most commonly used subjective measure of drowsiness, rating subjects on a scale from 1–9 depending on their state of sleepiness. The subjects were asked to perform self-evaluation of their drowsiness level using the KSS twice during the experiment: once at the beginning, and once at the end of the MD session. After each session, subjects rated their current drowsiness level. The ratings of subjects who exhibited drowsiness were compared before and after the MD session, using *t*-tests to determine the level of significance.

### 3.6. Ground Truth Management Using Observational Analysis

Video recording during data collection served as a measure of ground truth in this study. Data prior to drowsiness-related events were considered to represent active- or alert-state data. Video data was continuously recorded for the whole MD session, and was synchronized with the physiological data acquisition device. This helped the rater to mark the drowsiness-related events accurately, without any information loss. Video marking was performed only on the data recorded in the MD session. Video marking served as an independent measure for detecting driver drowsiness, as in several previous studies [12,36]. Drowsiness-related events were identified based on a range of facial features, including eye blink duration, facial expressions, facial tone, eye blinking rate, and movements such as head-nodding and yawning. 

**Figure 2:**
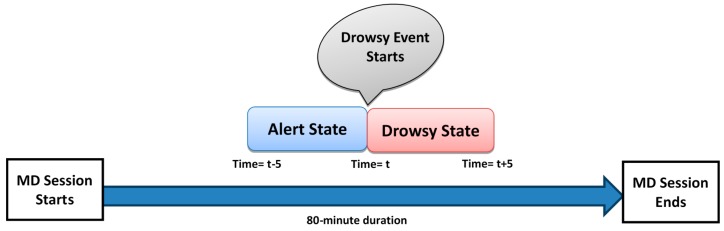
Drowsy and alert event marking process using video recordings from the MD session (t is in min).

Not all subjects exhibited signs of drowsiness, so only data from drowsy subjects were used for analysis. Eleven out of 22 subjects became drowsy during the experiment. The 5-min period prior to a drowsy event was used as data to represent the alert state, while the 5-min period after a drowsy event was used as data to represent the drowsy state, as shown in Figure 2. 

### 3.7. Physiological Data Pre-Processing

The EEG was pre-processed offline to remove muscle and eyeblink artifacts. Additionally, a band-pass filter of 0.5–40 Hz was used to remove the line noise and DC offset from the EEG and ECG data. Both the removal of eye blink artifacts and filtering were performed using the BESA software (MEGIS Software GmbH, Gräfelfing, Germany). Finally, EEGLAB [38] software was used for visual inspection of the physiological data, which was aligned with the video recording to remove movement artifacts and other unwanted signals produced from head or body movements.

### 3.8. Feature Extraction of Physiological Data

#### 3.8.1. EEG Time Domain Feature Extraction

Raw data acquired from physiological signals cannot be applied directly to machine learning algorithms for classification purposes because it is not meaningful (i.e., lack of discriminate properties), making feature extraction an essential stage. Therefore, various statistical features (mean, variance, minimum, maximum, energy [6]) and complexity measure (sample entropy [4]) were derived from the time domain EEG signal. The mathematical expressions to compute energy and sample entropy are formulated in Equations (1) and (2), respectively:

(1) $$\mathrm{Energy}\left(E\right)=\overset{N}{\underset{i=1}{\sum }}{\left(\left|x_{i}\right|\right)}^{2}$$

(2) $$\mathrm{SampleEn}\left(m,r,N\right)=-\ln\frac{B^{m}\left(r\right)}{A^{m}\left(r\right)}$$

where $A^{m}\left(r\right)=\frac{1}{N-m}\sum_{i=1}^{N-m}A_{i}^{m}\left(r\right)$ and $B^{m}\left(r\right)=\frac{1}{N-m}\sum_{i=1}^{N-m}B_{i}^{m}\left(r\right)$. $B^{m}\left(r\right)$ gives the probability of two sequences which match for m points and m + 1 gives the probability to match for m + 1 points, respectively. Sample entropy is a very important feature since it quantifies the complexity present in the time domain signal. Therefore, it can be useful for non-linear analysis of EEG signal. For the calculation of SampleEn, the parameters chosen were *r = 0.2 × standard deviation*, *m = 2* and *N* is the number of data points.

The sampling frequency of the signal was 500 Hz and window size of 1000 samples (N) was used with 50% overlap. Therefore, an analysis of 60 s data yielded 60 points. Later on, all time and frequency domain features derived from EEG were averaged across a minute providing a single value per min.

#### 3.8.2. EEG Frequency Domain Feature Extraction

Time domain analysis of EEG data does not provide detailed information about the activation of brain regions during a particular activity (i.e., which brain regions are activated during drowsiness, which frequency bands are activated in drowsiness) and their association with human physiological states. Therefore, we performed frequency domain analysis of the EEG data, which includes absolute power and relative power across all EEG channels and frequency bands. 

The frequency range was divided into frequency bands: Delta (0.5–4 Hz), Theta (4–8 Hz), Alpha (8–12 Hz), Beta (12–30 Hz) and Gamma (1–40 Hz) [28]. Absolute power was computed for all frequency bands (i.e., Delta power-$P_{\delta -abs}$, Theta power-$P_{\theta -abs}$, Alpha power-$P_{\alpha -abs}$, Beta power-$P_{\beta -abs}$, Gamma power-$P_{ᵞ-abs}$) across each electrode (5 frequency bands × 19 electrodes). Thus, for each alert and drowsy states, 95 features were obtained. In a similar way, relative power was computed for all frequency bands ($P_{\delta -rel},P_{\theta -rel},P_{\alpha -rel},P_{\delta -rel},P_{ᵞ-rel}$) using the following Equations (3)–(7):

(3) $$P_{\delta -\mathrm{rel}}=\frac{P_{\delta -\mathrm{abs}}}{P_{\mathrm{total}\_\mathrm{power}}}$$

(4) $$P_{\theta -rel}=\frac{P_{\theta -abs}}{P_{total\_power}}$$

(5) $$P_{\alpha -rel}=\frac{P_{\alpha -abs}}{P_{total\_power}}$$

(6) $$P_{\beta -rel}=\frac{P_{\beta -abs}}{P_{total\_power}}$$

(7) $$P_{ᵞ-rel}=\frac{P_{ᵞ-abs}}{P_{total\_power}}$$

Relative power is very important feature since it describes the contribution of the power at a particular frequency band to the overall EEG power ($P_{total\_power}$).

#### 3.8.3. ECG Data Feature Extraction

Power analysis of the HRV signal, derived from the raw ECG signal was also performed and divided into three frequency bands; very low frequency (VLF) (below 0.04 Hz), low frequency ($P_{LF}$) (0.04–0.15 Hz), and high frequency ($P_{HF}$) (0.15–0.4 Hz) [39]. The ratio of $P_{LF}$ to $P_{HF}$ i.e., $R_{LF-HF}$ was also computed, providing a measure of the sympathovagal balance of the body. The measures: $P_{LF}$, $P_{HF}$ and $R_{LF-HF}$ represent normalized power obtained from the absolute power using Equations (8)–(10):

(8) $$P_{LF}=\frac{P_{LF-abs}}{P_{Total}-P_{VLF-abs}}$$

(9) $$P_{HF}=\frac{P_{HF-abs}}{P_{Total}-P_{VLF-abs}}$$

(10) $$R_{LF-HF}=\frac{P_{LF}}{P_{HF}}$$

where $P_{VLF-abs},P_{LF-abs}$, $P_{HF-abs}$ represent the absolute powers of; very lower frequency, low frequency and high frequency bands respectively. The study of normalized power of HRV signal is very important since it helps to study the balance behavior between the sympathetic and parasympathetic nervous activity. Each of the features presented in Equations (7)–(9): $P_{VLF-abs}$, $P_{LF}$, $P_{HF}$ and $R_{LF-HF}$ were computed across a sliding time windows of 5 min with 80% overlap, thus providing a single value every minute [40]. 

### 3.9. Feature Selection

The feature-set extracted from physiological signals may contain redundant information (features that are unable to differentiate between alertness and drowsiness), which can lead to performance degradation, when applied directly to the classifier. Therefore, all the feature sets were subjected to a feature selection process prior to the classification stage. This was achieved using paired *t*-test to identify only the statistically significant features (*p* < 0.05) that can discriminate between the alert and drowsy states among all subjects. Before conducting the *t*-test, we checked that the data were normally distributed using the Kolmogorov-Smirnov test [41], since it is prerequisite before applying *t*-test.

### 3.10. Classifying Drowsiness and Channel Reduction

ECG and EEG features showing significant drowsiness-related differences were applied independently, and together, to the support vector machine (SVM) classifier (Pearson VII function-based universal kernel) using leave-one-subject-out-cross-validation procedure. The classification was performed using the Weka data mining software (Version 3.6.12, University of Waikato, [42]). Performance analysis of various combinations of EEG channels with ECG was also performed, to investigate the effects of reducing the number of EEG channels.

## 4. Results and Discussion

### 4.1. Subjective Measures

**Figure 3:**
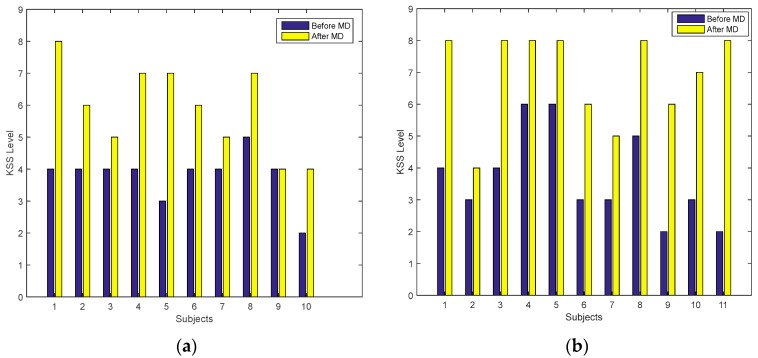
KSS rating of each subject before and after MD session in: (**a**) Alert group; (**b**) Drowsy group.

KSS rating levels at the start and the end of the MD session were analyzed to study the effects of drowsiness on subjective ratings. For this purpose, data was divided into two groups: the alert group (subjects who were alert throughout the driving session); and the drowsy group (subjects who exhibited drowsiness during the driving session). The KSS rating levels of both groups are shown in Figure 3a,b for the alert and drowsy groups, respectively. Figure 3b shows that the drowsy group exhibited higher KSS ratings at the end of the MD session compared with the start (before MD), when they were alert. However, the alert group (Figure 3a) also showed a similar pattern to the drowsy group (Figure 3b), comparing the data before and after MD. 

The *t*-test analysis (when applied on both groups independently, between the KSS levels before and after MD) shows that the KSS scale was significant (*p* < 0.05) in both groups (alert and drowsy) at the start and end of the MD session. Because this measure was significant in both groups during the experiment, it cannot be used as a discriminating measure for drowsiness. These findings indicate that subjective measures were not reliable for detecting drowsiness alone, and that solely relying on self-reported measures may not provide a meaningful measure of a person’s actual physiological state. 

### 4.2. Physiological Measures

#### 4.2.1. EEG Time Domain Analysis

Physiological data analysis was performed only for the 11 subjects who exhibited drowsiness, among the total sample of 22 subjects. Significant time domain features obtained from nineteen EEG channels after performing *t*-test are represented in Table 1 along with the *p*-values. 

Most commonly selected features were; energy, SmapleEn and maximum value. The signal energy varied from alert to drowsy state. Our findings regarding the EEG energy analysis are in agreement with the work by Mardi et al. [6] where the signal energy significantly changed in the occipital- and parietal brain regions when transitioning from alert to drowsy states.

Sample entropy feature was found to be a very useful in detecting driver drowsiness and showed statistical significances for channels; P4, O1 and O2 (Table 1). It decreased when transitioning from alert state to drowsy state indicating that the complexity of EEG signal decreases with the increase in drowsiness. These observations are in accord with [43], showing a decrease in sample entropy in occipital and parietal regions of the brain, as drowsiness progresses. 

#### 4.2.2. EEG Frequency Domain Analysis

**Figure 4:**
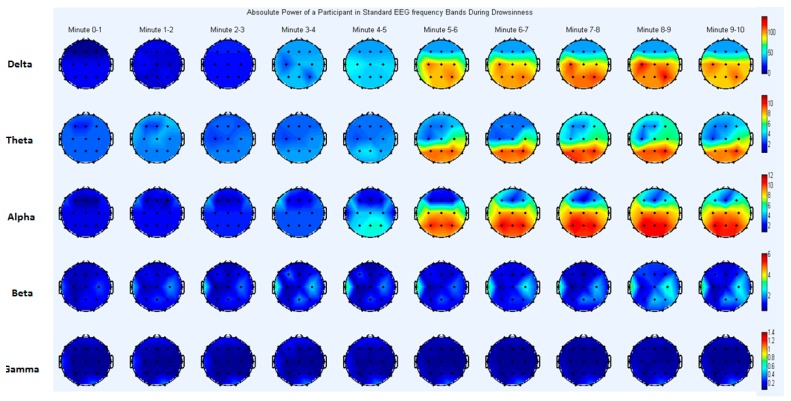
Brain topography of absolute power for standard EEG frequency bands obtained from the EEG data of one subject.

For better understanding of EEG frequency analysis, a topographical map is presented in Figure 4. The brain topography shows the absolute power for one subject, to visualize regional brain activation during the transitional phase from alertness to drowsiness. Data was divided into ten-min blocks; the first five min in each block represent the alert state of the subject, while the last five min represent the drowsy state (Figure 2). 

Absolute power and relative power were computed for all drowsy subjects (eleven) and for all EEG channels (nineteen) and frequency bands (delta, theta, alpha, beta, gamma). Table 2 shows only the features that exhibited significant differences (*p* < 0.05) after performing the paired *t*-tests to compare the alert and drowsy states.

##### Absolute Power Analysis

The analysis revealed that absolute power was significantly different between the drowsy and alert states (*p* < 0.05) in various frequency bands and brain regions (Table 2). Delta power increased significantly from alertness to drowsiness in the parietal (P4) and central (C3) regions (Table 2). In addition, theta power increased significantly in the parietal region (P4). Alpha power increased in many brain areas, including central (C3, Cz), occipital (O1, O2) and parietal (P3, P4 and P7) regions (see Table 2). The current findings of significant drowsiness-related differences in delta and theta band power are in accord with the results of a previous study [44], showing a significant increase in activation in the lower frequencies of the EEG signal over parietal and central brain regions during drowsiness. 

Alpha waves are primarily observed over the occipital and parietal regions, and are known to be activated during periods of relaxation and lower levels of mental activity, typically disappearing in the alert state, or when there is an increase in mental activity [45]. In addition, these waveforms are thought to be mainly generated by the visual cortex, located in the posterior part of the cerebrum. Our results indicate that, as drowsiness progressed, alpha band power increased significantly in the occipital and parietal regions of the brain. It should be noted that there has been some inconsistencies in previous findings, with some studies reporting variations only in the alpha band [46], and others reporting changes in the delta, theta and alpha bands [28,47]. However, these previous studies have primarily linked drowsiness with low-frequency activation, in line with the current findings.

##### Relative Power Analysis

Relative power analysis revealed that alpha power changed during the drowsy state (Table 2), and this increase in power was significant (*p* < 0.05) in the occipital (O1, O2) and parietal (P4, P8) regions of the brain. The results regarding the relative alpha power were in accord with those of a previous study [31], which reported that relative power in the alpha band increased significantly in the parietal region of the brain during the transition from the alert state to the drowsy state. As drowsiness increases, the vigilance level of a driver typically decreases, potentially leading to the loss of control of a vehicle. Vigilance is primarily related to activity in occipital and frontal brain regions. Activation of alpha power in occipital areas indicates low vigilance and increased drowsiness, supporting the notion that increased alpha power in the occipital region (O1, O2) is an indicator of drowsiness.

#### 4.2.3. ECG Results

The analysis of ECG data showed that the HRV features $P_{VLF-abs}$, $P_{LF}$, $P_{HF}$ and $R_{LF-HF}$ were significantly different between alert and drowsy states (paired *t*-test; *p* < 0.05). Although HR showed some variation in both the alert and drowsy states, the results revealed no statistically significant differences in HR between the two states. Some of the ECG data were published in an earlier study [48], but this is the first report of the performance analysis (using the machine learning algorithm) and its integration with EEG data. Table 3 shows the results from 11 subjects in the alert and drowsy states, in normalized units. 

Spectral analysis of HRV is commonly used to examine the activity of the sympathetic and parasympathetic nervous systems [49]. The low frequency component of HRV is influenced by both the sympathetic and parasympathetic activity while high frequency component is only linked with parasympathetic activity [50]. An increase in sympathetic activity is associated with an increase in $P_{LF}$, and an increase in parasympathetic activity is associated with an increase in HF power. Our analysis of the low frequency component $P_{LF}$ revealed a significant decrease from the awake to the drowsy state. This decrease in $P_{LF}$ indicates that subjects were in a transitional phase from alert state to drowsy state, and sympathetic activity decreased with the increase in drowsiness. In contrast, the $P_{HF}$ component exhibited a significant increase when the subject transitioned from a wakeful state to a drowsy state, and parasympathetic activity increased with the onset of drowsiness. This result is in accord with [51] that parasympathetic activity increases during the transition from the alert state to the resting or drowsy state. $R_{LF-HF}$ was also analyzed in this study to find the relationship between sympathetic and parasympathetic activity. Our analysis revealed that the $R_{LF-HF}$ ratio decreased in the drowsy state compared with the alert state. This result is in line with previous findings [36,52] reporting that an increase in drowsiness results in a decrease in $R_{LF-HF}$. This measure also indicates that drivers were losing control and attention. 

### 4.3. Performance Analysis by Combining EEG and ECG Channels Compared with Using Them Alone

One of the objectives of this study was to combine the significant features extracted from different physiological signals to improve the performance of the driver drowsiness detection system. To this end, we first assessed the performance of EEG and ECG separately. We then combined significant features using SVM to determine the effects of using both types of physiological signal on the performance of the drowsiness detection system. The significant features obtained from the EEG (Table 1 and Table 2) and ECG (Table 3) features used as physiological measures were applied to the SVM classifier. A confusion matrix and performance metrics were obtained from ECG and EEG separately, then combined (Table 4).

The performance metrics reported in Table 4 show that the classification accuracy achieved by ECG features was 70%, the accuracy achieved by all EEG features was 76.36%, and the achieved by combining of EEG and ECG features was 80.90%. These findings give rise to the fact that combining the proposed feature set of physiological signals significantly improves the system’s performance.

### 4.4. Channel Reduction to Improve Wearability

Another objective of the study was to test the effects of reducing the number of EEG sensors involved in the system, to increase the comfort level for the driver and make the system more practical for use in real driving conditions. In ECG data collection, the optimum electrode number achieved was one (i.e., a single electrode applied to the bottom of the neck was sufficient for acquiring the ECG signal). Although the feature selection process using the statistical test reduced the number of channels from 19 (see Table 1 and Table 2), there is a benefit to reducing the number further, because wearing even four or five EEG electrodes would still be likely to interfere with driving performance. An analysis of the significant features obtained from the EEG signal revealed that six channels were frequently selected by the statistical test showing significant differences between the alert and drowsy states. The channels were: C3, P4, P7, O1 and O2. Thus, we performed channel selection, in which significant features extracted from different combinations of six EEG channels were used as inputs to the classifier along with the significant features extracted from a single ECG channel. Table 5 shows the performance of the drowsiness detection system when significant EEG channels were combined. 

Using ECG alone as an indicator of drowsiness resulted in accuracy of 70%. Importantly, the results in Table 5 show that a single EEG channel (specifically either O2 or P7) was able to detect driver drowsiness effectively, when combined with an ECG channel, providing more than 80% accuracy (highlighted with bold text in Table 5). Therefore, we propose that two channels (i.e., one EEG channel and one ECG channel) are sufficient enough to provide an acceptable level of performance around 80% [27] and a system that is more feasible in real-world driving conditions. Although, Lin et al. [26] have made an effort to reduce the number of EEG electrodes, their approach is different than our method in several ways: 1) in-depth analysis of all EEG electrodes in 10–20 systems in our method, instead of using only occipital region for analysis as a starting point by Lin et al.; 2) analyzing and combining variety of features from EEG and ECG in our approach, instead of using only features from EEG by Lin et al.; 3) performance comparison of several electrode combinations from different brain regions (Table 5) for better understanding, instead of using only occipital region by Lin et al.

The findings regarding the use of single EEG channel with ECG are quite important as these can lead to the development of drowsiness detection devices which will perform decisions in real time, based on the cognitive state of the driver. The potential future device will have a built-in training model of drowsiness detection and can produce alerts in real-time. For this purpose, the classifier training model will be built on the dataset utilized in this study and the testing dataset will be acquired in real driving conditions to enable decision making in real time (alert/drowsy). One of the challenges is to perform pre-processing of EEG signal in real time before feature computation. In this regard, some authors [53,54] have tried to automatically remove the EEG artifacts. Other challenges include reducing the computational complexity of feature extraction stage in order to make it feasible in real-time. 

## 5. Conclusions and Future Work

The study in our paper investigated the use of features extracted from ECG and EEG signals for the detection of driver drowsiness. The experiment was performed with a simulator-based driving environment, with a sample of 22 subjects. A range of EEG features were extracted, including time domain statistical and complexity measures, frequency domain absolute and relative powers. Moreover, HR and HRV features were extracted from ECG data during the drowsy and alert states, and validated with video data. Features obtained from EEG time domain analysis were energy and entropy exhibiting statistical significance in occipital and parietal brain regions. Furthermore, EEG frequency domain analysis showed significant drowsiness-related changes in absolute power in the delta, theta and alpha bands in the central, parietal and occipital regions. Relative power showed significant changes in the alpha band in occipital and parietal regions. The results revealed a significant increase in both relative and absolute power during the transitional phase from the alert state to the drowsy state. All of the significant features obtained from EEG and ECG analysis were applied to the SVM classifier to evaluate system performance. The proposed combination of ECG and ECG features achieved a performance of 80.90%, highlighting the fact that combining physiological signals improves the system’s performance instead of using them alone (EEG or ECG).

Although EEG is a reliable indicator of driver drowsiness, existing EEG systems are impractical in real driving conditions, because of their limited wearability. Therefore, reducing the number of EEG electrodes in combination with ECG recording has the potential to provide a solution that overcomes these limitations. We tested the effect of reducing the number of EEG electrodes, revealing that an acceptable level of performance could be achieved with only two electrodes (one EEG and one ECG). This finding may be useful for the development of more wearable and practical drowsiness detection systems for use in real driving conditions. Based on these results, we propose three recommendations for future research:

- The study was conducted in a simulator-based environment in which the room temperature, lighting conditions and other environmental variables were constant. However, in real driving conditions these variables constantly change, introducing more artifacts into the physiological signals due to diverse environmental conditions and driving behavior. Therefore, future studies should examine the performance of this system under real driving conditions with variable environmental conditions.
- To develop a real time drowsiness monitoring device, the computational complexity of the proposed system should be decreased. This includes the optimization of the drowsiness detection algorithm and the removal of various artifacts in real time.
- The effects of drowsiness should be further explored using other physiological signals such as electrooculogram (EOG) and electromyogram (EMG). EMG sensors are less limited in terms of wearability compared with EEG sensors, which require drivers to wear a large cap while driving.

## Figures and Tables

**Table 1:** Significant features obtained from the EEG time domain analysis.

Channel	Significant Feature (*p*-Value)
P4	Var(0.020), Energy(0.001), Min (0.022), Max(0.016), SampleEn (0.013)
P7	Max (0.018), Energy (0.005)
C3	Energy(0.016)
O1	Var (0.039), Max(0.027), Energy (0.009), SampleEn(0.044)
O2	SampleEn(0.030)

**Table 2:** Significant features obtained from the EEG power analysis.

Channel	Significant Feature (*p*-Value)
P3	$P_{\alpha -abs}\left(0.048\right)$
P4	$P_{\delta -abs}\left(0.023\right)$, $P_{\theta -abs}\left(0.037\right)$, $P_{\alpha -abs}$ (0.014), $P_{\alpha -rel}\left(0.014\right)$
P7	$P_{\alpha -abs}$ (0.044)
P8	$P_{\alpha -rel}$ (0.023)
C3	$P_{\delta -abs}$ (0.006), $P_{\alpha -abs}\left(0.006\right)$
Cz	$P_{\alpha -abs}$ (0.032)
O1	$P_{\alpha -abs}$ (0.048), $P_{\alpha -rel}\left(0.030\right)$
O2	$P_{\alpha -abs}$ (0.039), $P_{\alpha -rel}$ (0.047)

**Table 3:** HRV features of each subject in alert and drowsy state.

State →	Alert State				Drowsy State			
Features →	$P_{LF}$ ^b^	$P_{HF}$ ^a^	$R_{LF-HF}$ ^b^	$P_{VLF}$ ^a^	$P_{LF}$ ^b^	$P_{HF}$ ^a^	$R_{LF-HF}$ ^b^	$P_{VLF}$ ^a^
Mean	0.54	0.32	2.01	859.82	0.46	0.37	1.39	1338.47
STD	0.10	0.08	0.98	114.12	0.08	0.06	0.59	121.61

^a^ Feature with significance level of *p* < 0.05, ^b^ feature with significance level of *p* < 0.01.

**Table 4:** Performance analysis by combining physiological signals.

**Accuracy**	**(a) Only ECG**		
70.00%	**Predicted Class**		
**Actual Class**	**Alert**	**Drowsy**	**← Classified as**
	39	16	**Alert**
	17	38	**Drowsy**
**Accuracy**	**(b) Only EEG**		
76.36%	**Predicted Class**		
**Actual Class**	**Alert**	**Drowsy**	**← Classified as**
	43	12	Alert
	14	41	Drowsy
**Accuracy**	**(c) Combining ECG and EEG**		
80.90%	**Predicted Class**		
**Actual Class**	**Alert**	**Drowsy**	**← Classified as**
	46	9	**Alert**
	12	43	**Drowsy**

**Table 5:** System accuracy using different combinations of EEG electrodes with ECG.

Channel Combination	Performance
O1, ECG	79.82%
**O2**, **ECG**	**80.90%**
**P7**, **ECG**	**80.00%**
P4, ECG	79.09%
C3, ECG	76.36%
